# Evaluation of upper limb lymphoedema and diagnostic accuracy of bioimpedance spectroscopy. A comprehensive validation in a Brazilian population

**DOI:** 10.3332/ecancer.2023.1649

**Published:** 2023-12-18

**Authors:** Fabíola C Brandini da Silva Tozzo, Almir José Sarri, Willian Eduardo Pirola, Uliana Basilio Cardoso da Silva, Marco Antonio de Oliveira, Cristiano de Pádua Souza, René Aloisio da Costa Vieira

**Affiliations:** 1 Post-Graduate Program, Barretos Cancer Hospital, São Paulo 14784-400, Brazil; 2 Department of Physical Therapy, Barretos Cancer Hospital, São Paulo 14784-390, Brazil; 3 Center of Epidemiology and Biostatistics, Barretos Cancer Hospital, São Paulo 14784-400, Brazil; 4 Department of Breast Cancer, Barretos Cancer Hospital, São Paulo 14784-400, Brazil; a https://orcid.org/0000-0002-7804-9053; b https://orcid.org/0000-0001-9184-584X; c https://orcid.org/0000-0003-3372-2504; d https://orcid.org/0009-0003-0162-0615; e https://orcid.org/0000-0001-6879-2778; f https://orcid.org/0000-0002-6412-8041; g https://orcid.org/0000-0003-2014-9016

**Keywords:** breast neoplasm, lymphoedema, diagnosis, biolectric impedance, ROC curve

## Abstract

Lymphoedema is a complication of breast cancer treatment. Its early diagnosis is related to a good prognosis for lymphoedema treatment. The bioimpedance spectroscopy (BIS) evaluates changes in extracellular fluid. The objective of our study was to evaluate the validity, agreement and accuracy of BIS in the diagnosis of breast cancer-related lymphoedema in a Brazilian population.

**Methods:**

This is a prospective, cross-sectional study of a convenience sample of 462 women who underwent surgical treatment for breast cancer (mastectomy or breast-conserving treatment). The validity, agreement and accuracy were performed comparing BIS (lymphoedema index (L-DEX) ≥ 6.5 or 10) with volumetry by water displacement, which is the gold standard for evaluating lymphoedema. Receiver operating characteristic curve was performed. Additionally, other methods like perimetry and indirect volumetry of the upper limbs were compared with water displacement volumetry (direct volumetry), and the BIS were compared with subjective evaluation.

**Results:**

Considering L-DEX ≥ 10 the sensitivity of the BIS was 44.1%, specificity 95.4%, positive predictive value (PPV) was 70.7%, negative predictive value (NPV) was 87% and kappa was 0.459. The BIS with L-DEX ≥ 6.5, the sensitivity, specificity, PPV, NPV and kappa were 57%, 88.5%, 55.8%, 89% and 0.452, respectively. Area under curve was 0.724 and a possible cut-off point of L-DEX ≥ 7.35 with sensitivity of 57%, specificity of 90.7% and kappa value = 0.489.

**Conclusion:**

Although BIS was significantly associated with the subjective evaluation of lymphoedema, it showed low sensitivity and agreement and moderate correlation when used as a method for diagnosing the condition. Thus, it is not the most valid method for evaluating lymphoedema. In addition, it was not the most accurate method when compared with other objective evaluation tools. Public health resources are scarce and must be used consciously. The knowledge that BIS is not a more accurate method than other, lower-cost instruments allows for better targeting of these resources.

## Background

Upper limb lymphoedema secondary to breast cancer treatment is a disabling, chronic and incurable sequelae caused by lymphatic insufficiency. Its incidence, at 12 months, is approximately 3% in patients who undergo sentinel lymph node (SLN) biopsy and 20% in patients who undergo axillary lymphadenectomy [1, 2]. Breast cancer-related lymphoedema (BCRL) is a time dependent event [3] and its prevalence ranges from 6% to 49%, and the cumulative incidence over 10 years is 41.1% [1, 2]. The main factors associated with the risk of lymphoedema secondary to breast cancer are axillary lymphadenectomy, radiotherapy in the drainage chain, including the supraclavicular fossa, mastectomy, presence of affected lymph nodes, body mass index (BMI), trauma and infection in the arm [3].

The clinical diagnosis of BCRL is based on the report of a patient who often complains of a swollen arm, weight gain and difficulty moving; however, 18% of patients with BCRL are asymptomatic [4]. The diagnosis can be complemented by different objective methodologies, such as volumetry, perimetry, perometry and bioimpedance spectroscopy (BIS) of the upper limb [4].

Volumetry, considered the gold standard for the evaluation of lymphoedema, can be assessed directly by water displacement (Archimedes principle), that is, the volume of water displaced is equal to the volume of the submerged object [5]. Another way of assessing lymphoedema by volumetry is indirectly by the frustum formula. Assuming that the upper limb can be approximated as a cone, the volume can be calculated, and one side can be compared with the other, thus measuring the difference in volume between them. Using the formula of a cylindrical cone [*V* = *h* (*C*_1_^2^ + *C*_1_*C*_2_ + *C*_2_^2^) / 12*π*] [6, 7] and evaluating 10 points, best accuracy was associated with 96.7 mL [8]. Lymphoedema is also defined as a difference in circumference of the affected arm ≥2 cm compared to the unaffected side at one or more points [9, 10]. Another way to evaluate the volume of the limb is the perometer, a device that calculates the volume of the limb by means of infrared lamps inserted in a square frame. This optical scanner is moved along the extended limb, and the limb’s volume is calculated using the shadows of the limb projected on this frame [11]. According to Levenhagen *et al* [12], its diagnostic property is not superior to other forms of evaluation, and the device is expensive, difficult to acquire and bulky.

The BIS measures the resistance of the extracellular fluid to the flow of an electric current through the tissues of the body, generating a score called the lymphoedema index (L-DEX). L-DEX is the ratio of the impedance of the extracellular fluid of the unaffected limb to that of the affected limb. The greater the volume of extracellular fluid, the lower the impedance to current flow and consequently the higher the L-DEX; thus, it has been used in the early detection of lymphoedema and monitoring of sequelae [13–15].

Comparing the multiple methods, the tape measure is the easiest method, and volumetry is the most reliable method, but it is necessary to build an adapted container, as proposed by Lette [16]. In the frustum method, several measurements of the limb are needed, associated with the use of formulas calculated using programs or a preconfigured spreadsheet [17]. Bioimpedance spectroscopic analysis is considered the most accurate method [13, 14], but it is associated with high costs.

Other equipment or imaging methods have also been considered in the characterisation of lymphoedema, such as computed tomography, magnetic resonance imaging, lymphoscintigraphy and tonometry. However, due to a lack of evidence, high costs or invasiveness, these tests are not recommended in clinical practice [12]. Lymphoscintigraphy is also considered an accurate gold standard, but it is not available for routine screening due to logistics and cost concerns [18].

It is important that the diagnosis of BCRL be performed as soon as possible since it is directly related to the success of the treatment of lymphoedema, since the disease has negative impacts on patient functionality and quality of life. The bioimpedance spectroscopic is a device that is being studied for the evaluation of lymphoedema, as it evaluates changes in extracellular fluid. To date, no studies have evaluated the validity of the BIS as a tool for assessing lymphoedema in the Brazilian population who have undergone treatment for breast cancer.

The objective of our study was to evaluate the validity, agreement and accuracy of BIS in the diagnosis of BCRL in a Brazilian population, comparing it with water displacement volumetry (direct volumetry).

## Materials and methods

This is a prospective, cross-sectional study of a convenience sample of 462 women who underwent surgical treatment for breast cancer (mastectomy or breast-conserving treatment) at the Women’s Outpatient Clinic of the Barretos Cancer Hospital (HCB) – Pio XII Foundation, from May 2015 to January 2021.

The inclusion criteria for participation in the study were as follows: previous surgical treatment of the breast; surgical treatment of the axilla, i.e., axillary lymphadenectomy or SLN biopsy; completion of radiotherapy in a period equal to or greater than 12 months; and an Eastern Cooperative Oncology Group score of 0 and 1. Participants were not included if they had metastatic disease, bilateral breast cancer, lymphoedema involving large volumes (circumference greater than 47.5 cm) that prevented evaluation with direct volumetry, or cardiac implants (pacemaker or defibrillator), were undergoing chemotherapy, or were pregnant.

The present study was approved by the Research Ethics Committee of the Pio XII Foundation – HCB and is registered under number 782/2014 and CAAE 28140214.1.0000.5437. All patients were included in the study only after acceptance of and signing an informed consent form.

### Sample

The incidence of BCRL is approximately 3% in patients who undergo SLN biopsy and 20% in patients who undergo axillary lymphadenectomy [2]. To reduce sampling bias, patients were selected at a ratio of one SLN biopsy for every three lymphadenectomies. The sample size was calculated according to the sensitivity and specificity of BIS in the detection of lymphoedema, according to the expressions suggested by Buderer [19], and performed using the online calculator available at https://wnarifin.github.io/ssc/sssnsp.html, accessed on 03/02/22.

For that calculation, the expected sensitivity (50.8%) and specificity (94%) values and prevalence of lymphoedema (21.3%) obtained in an interim sample were considered. A confidence level of 95% was also assumed for the confidence interval with a precision of ±2%, thus yielding a sample size of 461 participants.

### Assessment instruments

The participants underwent lymphoedema evaluation by BIS, volumetry, perimetry and self-report. Water displacement volumetry (direct volumetry) is the method adopted as the gold standard. In addition, sociodemographic data (age, weight, height, education and dominant limb) clinical data (date of initiation of treatment, pathological diagnosis, type of breast surgery, type of axillary surgery, data on radiotherapy and treated side) were collected. Weight in kilograms (kg) and height in metres (m) were obtained to calculate the BMI according to the formula: BMI = weight / (height × height).

### Bioimpedance spectroscopy

**Figure 1.**
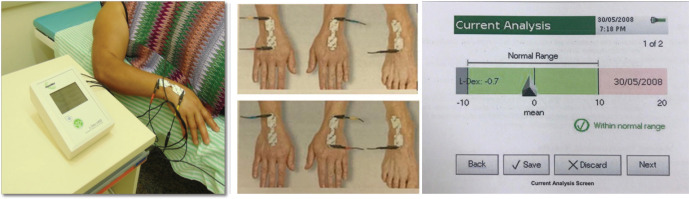
Bioimpedance spectroscopic. (a): Bioimpedance spectroscopic device ImpediMed, model U400. (b): Demonstration of the positioning of the electrodes on the hands and feet. (c): Demonstration of the spectroscopic bioimpedance analysis with the L-DEX value.

BIS was performed using an ImpediMed U400 device (Figure 1a) following the manufacturer’s guidelines: the examination was conducted at least 12 hours after physical activity, 2 hours after caffeine ingestion and after the patient had emptied her bladder.

The patient was instructed to remove metal accessories and shoes and then lie down in the supine position on a stretcher. The electrodes were placed on the dorsum of the hands and right foot (Figure 1b) after the sites were cleaned with gauze moistened with 70% alcohol. Subsequently, bioimpedance measurements were performed. Lymphoedema was considered if L-DEX ≥ 10 [20] or L-DEX ≥ 6.5 [21, 22] (Figure 1c).

### Perimetry

Perimetry was performed using an inelastic tape measure. The patient was placed in a sitting position with the upper limb being measured in shoulder flexion at 90° [23]. Circumference was measured using a tape measure, and measurements were taken every 5 cm, starting at the cubital fossa in both arms, until reaching the axillary line and ulnar styloid process as closely as possible. A difference of 2 cm between the circumference of the treated limb and the untreated limb in at least one of the measurements was considered indicative of lymphoedema [24].

### Direct volumetry

Equipment manufactured by the Department of Clinical Engineering of the HCB, according to the instructions proposed by Lette [16], was used to evaluate the volume of the upper limbs by water displacement. With the patient positioned next to the equipment, she was asked to lower her arm at low speed up to the last marking made on her arm during perimetry, then to repeat the same procedure with the other limb. The water displaced by each arm was recorded using a millimetre-scale Becker container. Lymphoedema was defined as a difference in volume between the treated limb and the contralateral limb ≥200 mL [20, 22].

### Indirect volumetry

To calculate the indirect volume, the cylindrical frustum formula [V^HR^ = *h* (*C*_1_^2^ + *C*_1_*C*_2_ + *C*_2_^2^) / 12*π*] was used. This formula uses the circumferences measured at two points (*C*_1_ and *C*_2_) and the distance (*h*) between these two points to estimate the volume of the segment. In the present study, the circumference measurements were taken every 5 cm, the same as the circumference measurements for perimetry. The limb volume is the sum of the volume of each segment.

Although we observed that 200 mL volumetry represents 96.7 mL [8] related to cylindrical frustum, we used current literature for comparison, which considers the difference of volume of 200 mL [20, 22] or 10% (volume of the affected limb/volume of the contralateral limb) [23].

### Subjective evaluation

The subjective evaluation of lymphoedema by the patient was performed using three questions of the *Breast Cancer Treatment Outcome Scale (BCTOS)* questionnaire that refers to the domain of lymphoedema. The BCTOS evaluates the functional and cosmetic results after conservative breast cancer treatment. The questions regard the sensation of heaviness in the arm, swelling of the arm and adjustment of the sleeves of shirts. Patients are asked to compare the treated side with the contralateral side and graded the difference from 1 to 4, where 1 corresponds to no difference, 2 to slight difference, 3 to moderate difference and 4 to great difference [25]. Lymphoedema was considered to be any sensation of difference, whether slight, moderate or large.

### Statistical analysis

The study population was characterised using descriptive statistics, that is, the mean, standard deviation, minimum and maximum for quantitative variables and frequency for qualitative variables.

To evaluate the association of sociodemographic variables and clinical data with lymphoedema assessed by direct volumetry was used Pearson's chi-square test and for the variables that presented *p* < 0.1, multivariate logistic regression analysis was performed, with Wald's chi-square test considering *p*-value < 0.05, odds ratio (OR) and 95% confidence interval (CI 95%).

For assessing the validity of BIS and other lymphoedema evaluation methods (indirect volumetry and perimetry), sensitivity, specificity, positive predictive value (PPV), and negative predictive value (NPV) were calculated through comparison with direct volumetry (gold standard). To assess agreement, the kappa statistic was calculated.

The receiver operating characteristic (ROC) curve and the corresponding cut-off point (the point yielding the highest sensitivity and kappa values) were plotted.

To verify normality, the Kolmogorov‒Smirnov test was used. None of the data from the present sample conformed to a normal distribution. To analyse the bivariate correlation between BIS and direct volumetry, the nonparametric Spearman correlation coefficient and intraclass correlation coefficient (ICC) were used.

The methods were compared using the *area under the curve* (AUC), in which an AUC equal to 1 indicates perfect sensitivity and specificity, whereas an AUC = 0.5 indicates poor sensitivity and specificity [26]. AUCs were compared according to their confidence intervals. If the intervals did not overlap, there was a significant difference between the curves.

Pearson's chi-square test was used to assess the association BIS with the subjective assessments and BMI; and direct volumetry, indirect volumetry, perimetry and BIS with BMI.

IBM SPSS software v.25 was used for all calculations except for the comparison of the ROC curves, which was performed with Stata software v.14.0. The significance level considered was 5%.

## Results

### Sociodemographic and clinical characteristics

The population of this study consisted of 462 women. The mean age of the participants was 57 years (SD ± 9; minimum 25, maximum 87). Slightly more than a third had incomplete primary education (38.5%) or were obese with a BMI ≥ 30 (33.4%), and the majority were right-handed (96.1%). Half had breast cancer on the right side, as shown in Table 1.

Regarding the characteristics of breast cancer and its treatment, 392 (89.5%) participants had invasive ductal carcinoma, 373 (81.3%) underwent quadrantectomy, 265 (57.7%) underwent axillary lymphadenectomy and 88 (19.2%) underwent SLN biopsy that progressed to lymphadenectomy, for a total of 353 lymphadenectomies (76.9%). Most patients did not undergo radiotherapy to the supraclavicular fossa. There was a statistically significant association between lymphoedema and the clinical variables BMI, armpit surgery and supraclavicular radiotherapy, as shown in Table 1.

A multivariate analysis was performed with the variables BMI and armpit surgery. In the BMI classification into underweight, normal, overweight and obese there was no significance, however, when classified into obese and non-obese, obese patients have a 2.32-fold increased risk of developing lymphoedema. In relation to armpit surgery, performing lymphadenetomy increases the chance of having lymphoedema by 7.77 times, as shown in Table 2.

### Evaluation of lymphoedema

Three participants did not undergo direct volumetry because they had injuries in the upper limb. One patient had burn blisters, and two had cuts.

Regarding the objective assessment tools for lymphoedema, direct volumetry, indirect volumetry, perimetry, L-DEX ≥ 10 and L-DEX ≥ 6.5, lymphoedema was present in 93 (20.1%), 131 (28.4%), 141 (30.5%), 59 (12.8%) and 96 (20.8%) women, respectively, as shown in Table 3.

In the subjective evaluations of lymphoedema as described by the participants who regarded a mild to great difference between the limbs, of the 462 women, 196 (42.4%) felt a difference in arm weight, 167 (36.1%) felt a difference in the fit of their shirts and 163 (35.3%) felt a difference in arm swelling (Table 3).

### Validity and agreement of BIS

When comparing patients with lymphoedema diagnosed by direct volumetry with those diagnosed by BIS with L-DEX ≥ 10, BIS did not diagnose lymphoedema in 52 of the 93 patients with a volume difference ≥200 mL in the upper limbs, resulting in moderate agreement between the methods with a kappa value = 0.459.

When comparing patients with lymphoedema diagnosed by direct volumetry with those diagnosed by BIS with L-DEX ≥ 6.5, 40 of the 93 patients with lymphoedema had L-DEX < 6.5, resulting in moderate agreement with a kappa value = 0.452 (Tables 4 and 5).

For L-DEX ≥ 10, the sensitivity and specificity of BIS were 44.1% and 95.4%, respectively, while if L-DEX ≥ 6.5, the corresponding values were 57% and 88.5%, respectively. For the lower threshold, there was a slight increase in sensitivity; however, there was a decrease in specificity and a slight decrease in the kappa value, as shown in Table 5.

### Correlation of BIS and volumetry

When evaluating the correlation between BIS and volumetry using the ICC, we observed a moderate correlation with an *α*-Cronbach value of 0.703 (95% CI = 0.643–0.753).

### ROC curve and cut-off point

When performing ROC curve analysis between the BIS and the difference in volume, a good *AUC* was obtained (AUC = 0.724). For an L-DEX cut-off point 1.35, the sensitivity was 74.2% and the specificity was 66.9%, but the kappa value was 0.298. For an L-DEX cut-off point of 7.35, the sensitivity was 57%, the specificity was 90.7%, and the kappa value was 0.489 (Table 5).

### Comparison of different methods for evaluating lymphoedema

Comparing the sensitivity, specificity, PPV, NPV and kappa of the different objective evaluation methods for upper limb lymphoedema, BIS with L-DEX ≥ 10 or L-DEX ≥ 6.5 was the method, demonstrating the lowest sensitivity and kappa value, as shown in Table 5. Table 6 shows the AUCs of BIS, indirect volumetry and perimetry. Comparison of the AUCs via their confidence intervals (CI 95%) shows a significant difference between the corresponding ROC curves (*p* < 0.001).

### Subjective evaluation of lymphoedema and BIS

Table 7 shows the data on the association between the subjective assessment of lymphoedema and the BIS assessment. The frequency of patients with L-DEX ≥ 10 or L-DEX ≥ 7.35 was higher and statistically significant among patients with a large difference in the feeling of heaviness in the arm, a difference in the fit of the shirt sleeve and a difference in arm swelling. At L-DEX ≥ 6.5, the frequency was higher, statistically significant among patients with a large difference in the feeling of heaviness in the arm, a moderate difference in the fit of the shirt sleeve and a large difference in arm swelling. In L-DEX ≥ 1.35, the frequency was higher, statistically significant, only among patients who reported a large difference in arm swelling.

### Comparison of different lymphoedema assessment methods with BMI

BMI is a risk factor for lymphoedema. Table 8 shows the comparison of BMI stratified into underweight, normal weight, overweight and obese, with BIS, direct volumetry, indirect volumetry and perimetry. The prevalence of lymphoedema in obese people was statistically significant with the methodologies of direct volumetry (*p* < 0.001), indirect (*p* < 0.001) and perimetry (*p* < 0.001) and there was no statistically significant difference in BIS at any cutoff point.

The BIS was not significantly associated with BMI even when stratifying into non-obese and obese.

## Discussion

BIS has emerged in the literature as a promising tool in the diagnosis of lymphoedema secondary to breast cancer, allowing measurement of the ratio of extracellular fluid to total fluid in the affected limb [2, 27]. It is a less invasive, objective method, fast and capable of being completed with an average time of 2 minutes [2].

At an L-DEX cut-off point of 10, BIS demonstrated a sensitivity of 44.1%, specificity of 95.4%, PPV of 70.7% and NPV of 87%. Spitz *et al* [21] also evaluated the sensitivity, specificity, PPV and NPV of the L-DEX score in measuring lymphoedema in 395 women at risk for lymphoedema secondary to breast cancer and obtained values of 7.5%, 98.5%, 71.4% and 67.5%, respectively. Compared to those in our study, the sensitivity and NPV values are lower; however, the specificity and PPV values are similar.

Some studies found sensitivity values greater than 60%. Fu *et al* [26], the sensitivity was 66%, and the specificity was 95%. However, these metrics were evaluated for assessing discriminability between the group with lymphoedema (*n* = 42) and the group at risk for lymphoedema (*n* = 150) according to the hypothesis that women with breast cancer had L-DEX < 10 and women with lymphoedema had L-DEX > 10. There was no comparison with a gold standard methodology. Qin *et al* [28] compared BIS (L-DEX > 10) with indocyanine green lymphography (ICG) in 62 participants with primary or secondary lymphoedema of the upper and lower limbs in both sexes. Of these patients, 54 (83.9%) had secondary lymphoedema, and 35 (67%) had lymphoedema secondary to breast cancer. The sensitivity was 64%, and the specificity was 100% [28]. The specificity was 100% because only four patients had negative ICG lymphography and were also negative on BIS. They did not calculate the PPV and NPV because they had a large variation in prevalence due to the wide range of aetiologies of the diseases.

Barrio *et al* [29] evaluated L-DEX as a diagnostic tool by sensitivity of the method. Similar to our study used the BIS U400 from ImpediMed and direct water displacement volumetry as the gold standard. In the Barrio *et al* [29] study, the sensitivity was 92%; only 13 of the 186 participants had lymphoedema, and BIS diagnosed 12 of them.

For an L-DEX cut-off point of 6.5, there was an increase in sensitivity to 57% and NPV to 89%, but there was a decrease in the specificity value to 88.5% and PPV to 55.8%. Similar to our study, Spitz *et al* [21] showed an increased sensitivity and NPV to 55.6% and 69.6%, respectively, and a decreased specificity and PPV to 90.8% and 55.6%, respectively, with this cut-off point.

ROC curves and possible cut-off points were plotted in our study population. Seeking a cut-off point that maximises sensitivity and specificity, L-DEX ≥ 1.35 had a sensitivity of 74.2% and specificity of 66.9%. However, a PPV of 36.35% indicated a low proportion of patients among those who had L-DEX ≥ 1.35, which could lead to an overestimation of cases of lymphoedema, generating a negative impact on the lives of these women since it is the complication most feared after breast cancer treatment [30]. L-DEX ≥ 7.35 had the same sensitivity and NPV as L-DEX ≥ 6.5, but a slight increase in specificity and PPV. Fu *et al* [26] described L-DEX > 7.1 as the best cut-off point among women with lymphoedema versus those at risk for lymphoedema.

In the analysis of agreement between the methods with direct volumetry of lymphoedema, we used the kappa coefficient, with moderate agreement between L-DEX ≥ 10 or ≥6.5 and perimetry and substantial agreement between direct and indirect volumetry. We did not find studies in the literature that evaluated agreement by the kappa coefficient.

The agreement according to the ICC between BIS and direct volumetry showed a moderate correlation. Ferro *et al* [31] evaluated the correlation of the amount of intracellular, extracellular and total fluid, as predicted by BioBas InBody510 multifrequency BIS, with the indirect volume of the upper limb ipsilateral to breast cancer surgery in 27 Brazilian volunteers with lymphoedema. The correlations were moderate and positive (Pearson correlation coefficient = 0.60 in the intracellular fluid; 0.50 in the extracellular fluid and 0.60 in the total fluid); however, the authors did not take into account the difference between limbs, and compare total bioimpedance water with the volume of the limb on the contralateral side. Therefore, it cannot be said that this moderate correlation was due to lymphoedema. Our study included 462 participants with lymphoedema or who were at risk for lymphoedema. The diagnosis was based on the difference between the volume of the treated limb and that of the contralateral limb. In addition, the BIS U400 device from ImpediMed was used, which generates L-DEX, a linearised L-DEX, through the ratio of the impedance of the unaffected limb to that of the affected limb.

Fu *et al* [26] found a significant correlation (Pearson's coefficient = 0.44) between BIS (ImpXCA) and indirect volumetry. Czerniec *et al* [23] found a strong correlation (Lin concordance correlation = 0.89) between indirect volume (affected limb/unaffected limb) and BIS (SFB7).

The AUC of BIS as a continuous variable was 0.724 (95% CI 0.654–0.794). In the study by Smoot *et al* [32], the AUC of BIS as a continuous variable was 0.83 (95% CI 0.76–0.90), but the BIS device used was the SFB7. The AUC of BIS (ImpXCA) as a continuous variable in the study by Fu *et al* [26] was 0.941 (95% CI 0.907–0.976).

The highest AUC was obtained for L-DEX ≥ 6.5. The higher the AUC is, the more accurate the method. Among all the objective tools that we evaluated in the present study, the one with the greatest accuracy was perimetry, followed by indirect volumetry. Although BIS is an objective method and can be performed rapidly, the device is currently too expensive for widespread acquisition in Brazil.

Studies have evaluated the association of self-reported symptoms by patients with objective assessments [23, 33]. In a systematic review, the most prevalent symptoms in patients with lymphoedema were swelling and heaviness. Additionally, the symptoms of swelling now, heaviness now or in the past, arm firmness and temperature increase were associated with the presence of lymphoedema and found moderate correlation between swelling and firmness in the past with BIS [34]. Our study also found a statistically significant association between symptoms of arm weight, shirt sleeve adjustment and arm swelling with BIS, with an exception of L-DEX ≥ 1.35, which supports our conclusion that it is not the best cut-off point despite its high sensitivity. Another cross-sectional study, like ours, also found an association of three self-reported symptoms (swelling, heaviness and tightness) with BIS [33].

Shah *et al* [35] point out as risk factors for the development of BCRL: extension of axillary surgery, radiotherapy, chemotherapy with high rates and BMI. In the present study we also found an association of BCRL with axillary lymph dissection (lymphadenectomy), supraclavicular fossa radiotherapy and higher BMI. Chemotherapy has not been evaluated.

As well as in the study, Vicini *et al* [36], evaluated the association between BIS and BMI and no association was observed. However, in our study, in addition to BIS, we evaluated the association of other lymphoedema assessment methods (direct volumetry, indirect volumetry and perimetry) with BMI and found association in all methods, except BIS, even at different cutoff points. Possibly, obesity can alter the impedance to the passage of the electrical current from the BIS, and this could be a negative factor in the use of this device in this population, however, more studies need to be carried out to elucidate this issue.

HCB is a public tertiary oncologic hospital, specialised in cancer treatment. This makes screening with brief returns for early diagnosis of centralised lymphoedema in the hospital difficult. This practice is also performed in Brazilian public hospitals.

The main limitation of this study is a cross-sectional evaluation. As all patients had already undergone cancer treatment, a pretreatment L-DEX was not collected. Another limitation of our study is that we did not collect information on previous lymphoedema, such as the time of lymphoedema, whether the patient had undergone or was undergoing treatment, and the type of lymphoedema treatment performed.

## Conclusion

Although BIS was significantly associated with the subjective evaluation of lymphoedema, it showed low sensitivity and agreement and moderate correlation in the Brazilian population when used as a method for diagnosing the condition. In addition, it was not the most accurate method when compared with other objective evaluation tools.

## Conflicts of interest

The authors declare no conflicts of interest.

## Funding

This study was supported by Fundação de Amparo à Pesquisa do Estado de São Paulo (FAPESP) under the number 14/0819-0 and from the Teaching and Research Institute of the Cancer Hospital of Barretos, number IEP 5/2017.

## Informed consent

All patients were included in the study only after acceptance of and signing an informed consent form. The present study was approved by the Research Ethics Committee of the Pio XII Foundation – HCB and is registered under number 782/2014 and CAAE 28140214.1.0000.5437.

## Author contributions

Concept/idea/research design: FCBST, RACV, AJS and MAO; writing: FCBST, RACV, AJS, WEP and CPS; data collection: FCBST and UBCS; data analysis: FCBST, RACV and MAO; project management: FCBST and RACV; providing facilities/equipment: RACV; and consultation (including review of manuscript before submitting): FCBST, RACV, AJS, UBCS, WEP, MAO and CPS. All authors read and approved the final manuscript.

## Figures and Tables

**Table 1.** Sociodemographic factors and clinical data and the association with lymphoedema diagnosed by water displacement volumetry.

Variable	Category	*N*	%	Lymphoedema		*p*-value
				Positive *N* (%)	Negative *N* (%)	
Education	Illiterate	13	2.8	2 (18.2)	9 (81.8)	0.456
	Incomplete elementary school	178	38.5	28 (22.8)	95 (77.2)	
	Completed first degree	80	17.3	28 (23.1)	93 (76.9)	
	High school	114	24.7	11 (13.3)	72 (86.7)	
	Higher education	77	16.7	24 (19.8)	97 (80.2)	
BMI	Underweight (<18.5)	6	1.3	0 (0)	6 (100)	0.001
	Normal (18.5–24.9)	125	27.3	14 (11.25)	111 (88.8)	
	Overweight (25–29.9)	174	38.0	32 (18.4)	142 (81.6)	
	Obese	153	33.4	47 (30.7)	106 (69.3)	
Limb at risk	Right	231	50	54 (23.5)	176 (76.5)	0.104
	Left	231	50	39 (17)	190 (83)	
Dominant limb	Right	441	96.1	87 (19.7)	354 (80.3)	0.225
	Left	18	3.9	6 (33.3)	12 (66.7)	
Histology	DCIS	25	5.7	4 (16)	21 (84)	0.444
	Invasive ductal carcinoma	392	89.5	77 (19.6)	315 (80.4)	
	Invasive lobular carcinoma	17	3.9	4 (23.5)	13 (76.5)	
	Other	4	0.9	2 (50)	2 (50)	
Type of breast	Quadrantectomy	373	81.3	73 (19.6)	300 (80.4)	0.520^*^
surgery	Radical or modified mastectomy	62	13.5	14 (22.6)	48 (77.4)	
	Skin or nipple-sparing mastectomy	11	2.4	3 (27.3)	8 (72.7)	
	Simple mastectomy	9	1.9	1 (11.1)	8 (88.9)	
	Other	4	0.9	2 (50)	2 (50)	
Armpit surgery	SLN biopsy	106	23.1	4 (3.8)	102 (96.2)	<0.001
	SLN biopsy + lymphadenectomy	88	19.2	20 (22.7)	68 (77.3)	
	Lymphadenectomy	265	57.7	69 (26)	196 (74)	
Boost radiotherapy	Performed	309	67.6	25 (16.9)	123 (83.1)	0.217
	Not performed	148	32.4	68 (22)	241 (78)	
Supraclavicular radiotherapy	Performed	172	37.6	48 (16.8)	237 (83.2)	0.022
	Not performed	285	62.4	45 (26.2)	127 (73.8)	

BMI = Body mass index; DCIS = ductal carcinoma *in situ*; SLN = sentinel lymph node

* For this analysis, the SLN biopsy + lymphadenectomy groups were grouped with the lymphadenectomy alone group

**Table 2.** Multivariate analysis of the type of axillary surgery and armpit surgery.

Variable		OR	CI (95%)		*p*-value
Armpit surgery	SLN biopsy	Ref.	Ref.	Ref.	Ref.
	SLN biopsy + lymphadenectomy or lymphadenectomy alone	7.77	2.77	21.81	<0.001
BMI	Not obese	Ref.	Ref.	Ref.	Ref.
	Obese	2.32	1.44	3.74	<0.001
	Constant	0.031			

SLN = Sentinel lymph node; BMI = body mass index

**Table 3.** Numbers and percentages of patients with lymphoedema assessed objectively by direct volumetry, indirect volumetry, BIS and perimetry and subjectively by sensation of arm weight, shirt sleeve adjustment and arm swelling.

Variable	Category	Number	%
Direct volumetry	≥200 mL	93	20.1
	<200 mL	366	79.2
	Not performed	3	0.7
Indirect volumetry	≥200 mL	131	28.4
	<200 mL	331	71.6
Perimetry	≥2 cm	141	30.5
	<2 cm	321	69.5
BIS L-DEX	≥10	59	12.8
	<10	403	87.2
	≥6.5	96	20.8
	<6.5	366	79.2
Arm weight	No difference	266	57.6
	Slight difference	105	22.7
	Moderate difference	61	13.2
	Large difference	30	6.5
Shirt sleeve adjustment	No difference	295	63.9
	Slight difference	102	22.1
	Moderate difference	39	8.4
	Large difference	26	5.6
Arm swelling	No difference	299	64.7
	Slight difference	93	20.1
	Moderate difference	47	10.2
	Large difference	23	5

**Table 4.** Contingency table with numbers and percentages of patients diagnosed with lymphoedema using BIS with L-DEX ≥ 10 and L-DEX ≥ 6.5.

	Volumetria						
	<200 mL	≥200 mL	Total		<200 mL	≥200 mL	Total
BIS < 10	349 (76%)	52 (11.4%)	401 (87.4%)	BIS < 6.5	324 (70.6%)	40 (8.7%)	364 (79.3%)
BIS ≥ 10	17 (3.7%)	41 (8.9%)	5 (12.6%)	BIS ≥ 6.5	42 (9.2%)	53 (11.5%)	95 (20.7%)
Total	366 (79.7%)	93 (20.3%)	459 (100%)	Total	366 (79.7%)	93 (20.3%)	459 (100%)

BIS = Bioimpedance spectroscopy

**Table 5.** Sensitivity, specificity, PPV, NPV and kappa coefficient in the evaluation of lymphoedema by BIS, indirect volumetry and perimetry.

	BIS				Indirect volumetry		Perimetry
	L-DEX ≥ 10	L-DEX ≥ 7.35	L-DEX ≥ 6.5	L-DEX ≥ 1.35	Vol ≥ 200 mL	Vol dif. ≥ 10%	
Sensitivity (%)	44.1	57	57	74.2	65.6	58.1	84.9
Specificity (%)	95.4	90.7	88.5	66.9	96.3	97	83.1
PPV (%)	70.7	60.95	55.8	36.35	81.3	83.1	57.7
NPV (%)	87	89.2	89	91	91.7	90.1	95.7
Kappa	0.459	0.489	0.452	0.298	0.666	0.620	0.587
95% CI	0.35–0.56	0.39–0.59	0.35–0.55	0.21–0.38	0.58–0.75	0.53–0.71	0.53–0.71

**Table 6.** AUCs of objective evaluation methods and their comparisons.

Method	Value	AUC	CI (95%)	*p* value
BIS	L-DEX	0.724	0.654–0.794	<0.001
	L-DEX ≥ 10	0.688	0.637–0.739	
	L-DEX ≥ 7.35	0.715	0.662–0.768	
	L-DEX ≥ 6.5	0.718	0.664–0.771	
	L-DEX ≥ 1.35	0.681	0.629–0.734	
Indirect volumetry	Vol ≥ 200 mL	0.792	0.742–0.842	
	Vol dif. ≥ 10%	0.765	0.715–0.817	
Perimetry	≥2 cm	0.904	0.871–0.937	

**Table 7.** Numbers and percentages of patients reporting subjective evaluations of arm weight, shirt sleeve adjustment and arm swelling and associations of subjective evaluations with BIS and L-DEX cut-off points ≥10, ≥7.35, ≥6.5 and ≥1.35 (HCB-SP, April 2018 to January 2020).

Sense of difference	BIS		*p*-value	BIS		*p*-value	BIS		*p*-value	BIS		*p*-value
	<10	≥10		<7.35	≥7.35		<6.5	≥6.5		<1.35	≥1.35	
Arm weight												
None	244 (91.7)	22 (8.3)	0.003*	233 (87.6)	33 (12.4)	<0.001*	228 (85.7)	38 (14.3)	<0.001*	165 (62)	101 (38)	0.328
Slight	85 (81)	20 (19)		73 (69.5)	32 (30.5)		71 (67.6)	34 (32.4)		56 (53.3)	49 (46.7)	
Moderate	52 (85.2)	9 (14.8)		49 (80.3)	12 (19.7)		48 (78.7)	13 (21.3)		35 (57.4)	26 (42.6)	
Large	22 (73.3)	8 (26.7)		19 (63.3)	11 (36.7)		19 (63.3)	11 (36.7)		15 (50)	15 (50)	
Shirt sleeve adjustment												
None	272 (92.2)	23 (7.8)	<0.001*	255 (86.4)	40 (13.6)	0.001*	250 (84.7)	45 (15.3)	0.001*	179 (60.7)	116 (39.3)	0.625
Slight	82 (80.4)	20 (19.6)		74 (72.5)	28 (27.5)		72 (70.6)	30 (29.4)		58 (56.9)	44 (43.1)	
Moderate	29 (74.4)	10 (25.6)		26 (66.7)	13 (33.3)		25 (64.1)	14 (35.9)		20 (51.3)	19 (48.7)	
Large	20 (76.9)	6 (23.1)		19 (73.1)	7 (26.9)		19 (73.1)	7 (26.9)		14 (53.2)	12 (46.2)	
Arm swelling												
None	281 (94)	18 (6)	<0.001*	267 (89.3)	32 (10.7)	<0.001*	262 (87.6)	37 (12.4)	<0.001*	197 (65.9)	102 (34.1)	<0.001*
Slight	71 (76.3)	22 (23.7)		63 (67.7)	30 (32.3)		60 (64.5)	33 (35.5)		42 (45.2)	51 (54.8)	
Moderate	36 (76.6)	11 (23.4)		33 (70.2)	14 (29.8)		33 (70.2)	14 (29.8)		25 (53.2)	22 (46.8)	
Large	15 (65.2)	8 (34.8)		11 (47.8)	12 (52.2)		11 (47.8)	12 (52.2)		7 (30.4)	16 (69.6)	

BIS = Bioimpedance spectroscopy; ≥ = greater than or equal to; < = less than, % = percentage

* *p* < 0.05

**Table 8.** Comparison between BMI and lymphoedema according to direct volumetry, indirect volumetry, perimetry, BIS with L-DEX ≥ 10 and BIS with L-DEX ≥ 6.5.

	BMI				*p*-value
	Underweight (%)	Normal (%)	Overweight (%)	Obese (%)	
Direct volumetry					<0.001
With lymphoedema	0 (0)	14 (11.2)	32 (18.4)	47 (30.7)	
No lymphoedema	6 (100)	111 (88.8)	142 (81.6)	106 (69.3)	
Perimetry					<0.001
With lymphoedema	0 (0)	25 (20)	52 (29.5)	65 (42.2)	
No lymphoedema	6 (100)	100 (80)	124 (70.5)	89 (57.8)	
BIS ≥ 10					0.779
With lymphoedema	1 (16.7)	17 (13.6)	19 (10.8)	22 (14.3)	
No lymphoedema	5 (83.3)	108 (86.4)	157 (89.2)	132 (85.7)	
BIS ≥ 6.5					0.748
With lymphoedema	2 (33.3)	27 (21.6)	33 (18.8)	34 (22.1)	
No lymphoedema	4 (66.7)	98 (78.4)	143 (81.2)	120 (77.9)	
BIS ≥ 1.35					0.544
With lymphoedema	2 (33.3)	56 (44.8)	66 (37.5)	67 (43.5)	
No lymphoedema	4 (66.7)	69 (55.2)	110 (62.5)	87 (56.5)	
BIS ≥ 7.35					0.671
With lymphoedema	2 (33.3)	24 (19.2)	30 (17)	32 (20.8)	
No lymphoedema	4 (66.7)	101 (80.8)	146 (83)	122 (79.2)	
Indirect volumetry					<0.01
With lymphoedema	0 (0)	20 (16)	51 (29)	60 (39)	
No lymphoedema	6 (100)	105 (84)	125 (71)	94 (61)	

BMI = Body mass index; BIS = bioimpedance spectroscopy
